# Spontaneous Tumor Lysis Syndrome Revealing Advanced Ovarian Carcinoma: From Oncologic Emergency to Complete Pathologic Response

**DOI:** 10.7759/cureus.113241

**Published:** 2026-07-23

**Authors:** Josean M Rosado Rivera, Alejandra P Rivera Caro, Madeline Guerrero-Gonzalez, Eden Ocana-Vazquez, Cristina Horta Vargas, Milton Carrero Quiñones, Santa Merle

**Affiliations:** 1 Internal Medicine, Mayagüez Medical Center, Mayagüez, PRI; 2 Addiction Medicine, Central University of the Caribbean, Bayamón, PRI; 3 General Medicine, Mayagüez Medical Center, Mayagüez, PRI; 4 Hematology and Oncology, Mayagüez Medical Center, Mayagüez, PRI

**Keywords:** acute kidney injury, ascites, ca-125, complete pathologic response, cytoreductive surgery, hyperuricemia, oncologic emergency, ovarian carcinoma, rasburicase, spontaneous tumor lysis syndrome

## Abstract

Tumor lysis syndrome (TLS) is an oncologic emergency characterized by metabolic abnormalities resulting from rapid tumor cell breakdown. It is most frequently observed following cytotoxic therapy in hematologic malignancies and is rarely encountered spontaneously in solid tumors. Spontaneous TLS associated with ovarian carcinoma remains exceptionally uncommon.

A 53-year-old woman with no pertinent medical history presented with progressive abdominal distension, dyspnea, and oliguria. Laboratory evaluation demonstrated acute kidney injury with a serum creatinine of 4.09 mg/dL, accompanied by hyperuricemia, hyperkalemia, hyperphosphatemia, and hypocalcemia, fulfilling the Cairo-Bishop criteria for clinical TLS. Computed tomography (CT) revealed massive ascites and a 16.2 × 15 × 15 cm pelvic mass without evidence of obstructive uropathy. Ascitic fluid cytology demonstrated metastatic carcinoma. Tumor markers showed a cancer antigen (CA)-125 level of 445 U/mL and a CA-125:carcinoembryonic antigen ratio greater than 25, strongly supporting a gynecologic primary malignancy. In the absence of recent chemotherapy or other precipitating factors, spontaneous TLS secondary to previously undiagnosed ovarian carcinoma was diagnosed. The patient was treated with aggressive intravenous hydration, rasburicase, electrolyte correction, and close multidisciplinary monitoring, resulting in rapid normalization of metabolic abnormalities and recovery of renal function without dialysis.

Following stabilization, the patient underwent neoadjuvant carboplatin-paclitaxel chemotherapy followed by interval cytoreductive surgery. Histopathologic examination demonstrated a complete pathologic response, with no residual invasive carcinoma. Follow-up positron emission tomography/CT imaging showed no evidence of residual or metastatic disease, and CA-125 normalized to 5.2 U/mL.

This case highlights spontaneous TLS as a rare initial manifestation of advanced ovarian carcinoma and emphasizes the importance of early recognition and prompt intervention. Timely management may reverse life-threatening metabolic complications, facilitate definitive oncologic treatment, and result in favorable long-term outcomes.

## Introduction

Tumor lysis syndrome (TLS) is a potentially fatal oncologic emergency caused by rapid destruction of malignant cells, leading to the release of intracellular potassium, phosphorus, and nucleic acids into the systemic circulation [1]. The resulting metabolic derangements include hyperuricemia, hyperkalemia, hyperphosphatemia, hypocalcemia, acute kidney injury, cardiac arrhythmias, seizures, and death if not promptly recognized and treated [2]. TLS is diagnosed using the Cairo-Bishop criteria: laboratory TLS requires two or more of the following abnormalities occurring within three days before or seven days after cytoreductive treatment: uric acid ≥8 mg/dL, potassium ≥6 mEq/L, phosphorus ≥4.5 mg/dL, or calcium ≤7 mg/dL (or a 25% change from baseline for each parameter); clinical TLS additionally requires evidence of renal impairment, cardiac arrhythmia, or seizures [1,2].

TLS is most commonly observed after initiation of cytotoxic therapy in patients with highly proliferative hematologic malignancies, particularly acute leukemias and high-grade lymphomas [2,3]. In contrast, spontaneous TLS, occurring in the absence of anticancer therapy, is uncommon and has been reported only rarely in solid tumors [4-6]. Recognized risk factors for spontaneous TLS in solid tumors include high tumor burden, rapid cell proliferation, high chemosensitivity, elevated baseline lactate dehydrogenase and uric acid levels, preexisting renal insufficiency, and dehydration [4]. Among gynecologic malignancies, ovarian carcinoma represents an especially unusual setting for spontaneous TLS, with only a handful of cases reported in the literature to date [7], and current oncologic guidelines do not specifically address this presentation [8].

We report a case of spontaneous TLS as the initial manifestation of previously undiagnosed advanced ovarian carcinoma presenting with severe metabolic abnormalities and acute kidney injury. Early recognition and treatment resulted in complete renal recovery, allowing subsequent multimodal oncologic therapy that culminated in a complete pathologic response and complete metabolic remission.

## Case presentation

A 53-year-old woman with no pertinent medical history presented with several weeks of progressive abdominal distension, exertional dyspnea, reduced oral intake, and decreased urine output. She denied prior malignancy, recent chemotherapy exposure, or use of nephrotoxic medications.

**Figure 1:**
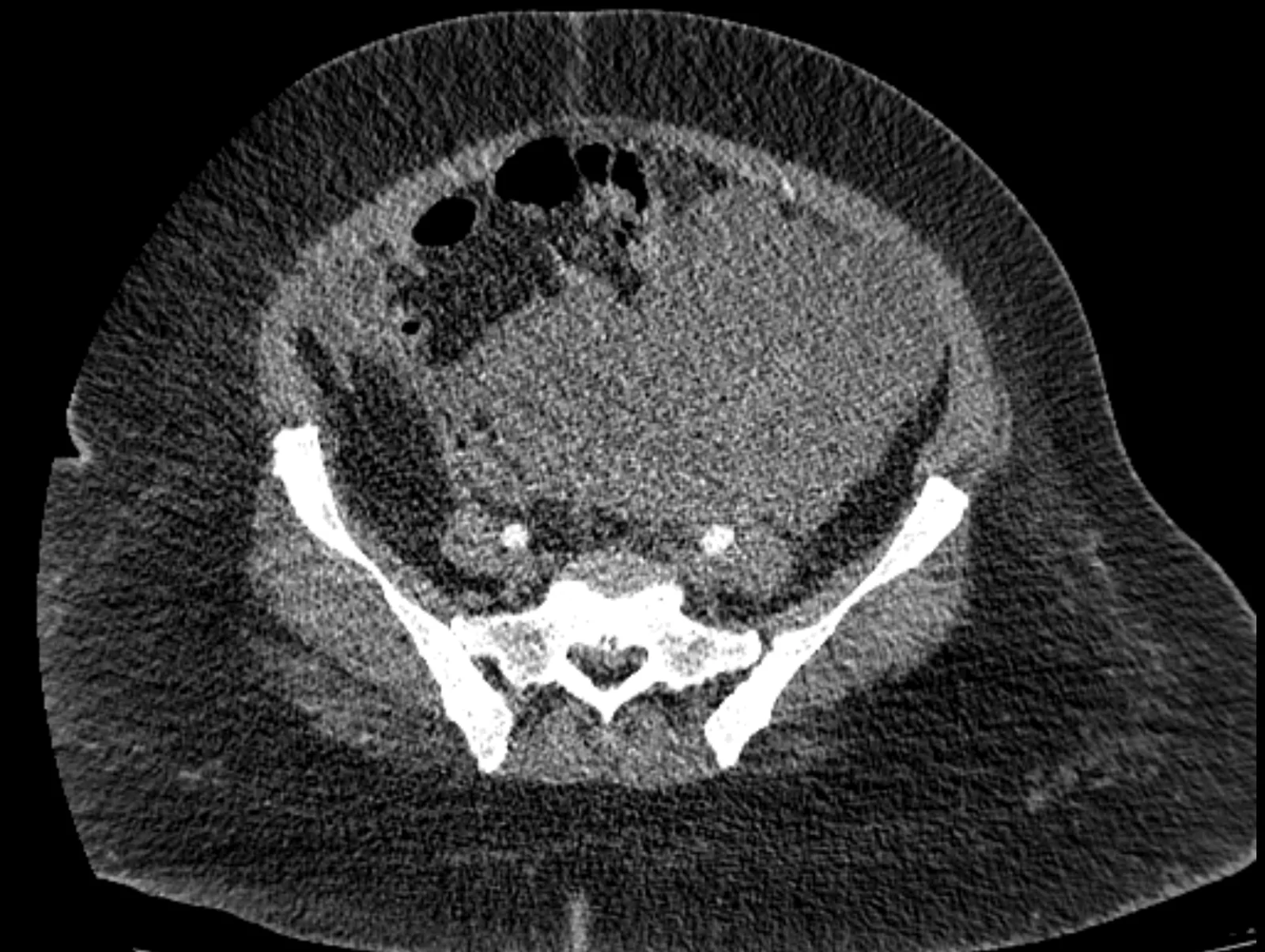
Contrast-enhanced CT of the abdomen and pelvis (coronal view), demonstrating a large heterogeneous pelvic mass measuring approximately 16.2 × 15 × 15 cm, with massive ascites CT: computed tomography

Physical examination revealed a markedly distended abdomen with tense ascites and preserved bowel sounds. No signs of peritonitis were present. Initial laboratory evaluation on admission (hospital day 1) demonstrated acute kidney injury with a serum creatinine level of 4.09 mg/dL, along with hyperuricemia (uric acid 12 mg/dL), hyperkalemia (potassium 6.1 mEq/L), hyperphosphatemia (phosphorus 8.7 mg/dL), and hypocalcemia (calcium 6.9 mg/dL), fulfilling all four Cairo-Bishop laboratory criteria for TLS (Table 1). Lactate dehydrogenase was 310 U/L. Renal ultrasonography demonstrated preserved renal size and architecture without hydronephrosis. Computed tomography (CT) of the abdomen and pelvis revealed massive ascites and a large pelvic mass measuring 16.2 × 15 × 15 cm, without evidence of urinary tract obstruction (Figure 1).

**Table 1:** Laboratory values at admission and after treatment ULN: upper limit of normal; LDH: lactate dehydrogenase

Parameter	Day 1 (admission)	Day 5 (posttreatment)	Cairo-Bishop threshold
Creatinine (mg/dL)	4.09	0.89	>1.5 × ULN
Uric acid (mg/dL)	12	2.6	≥8
Potassium (mEq/L)	6.1	4.2	≥6.0
Phosphorus (mg/dL)	8.7	3.3	≥4.5
Calcium (mg/dL)	6.9	8.3	≤7.0
LDH (U/L)	310	-	-

**Figure 2:**
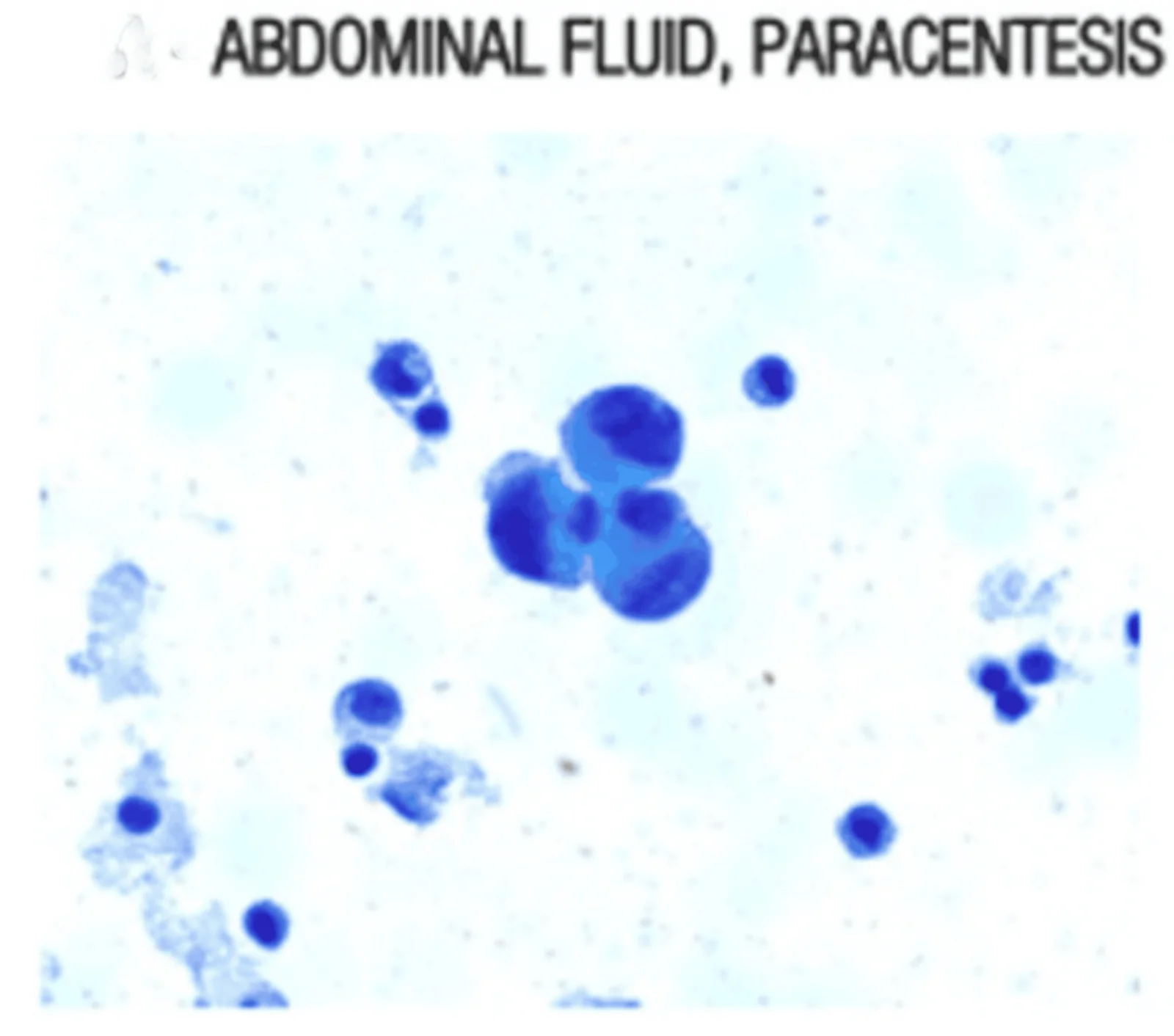
Ascitic fluid cytology demonstrating metastatic carcinoma consistent with an epithelial malignancy Ascitic fluid cell block preparation demonstrating clusters of atypical epithelial cells consistent with metastatic carcinoma. Immunoperoxidase stains performed on cell block preparations, with reactive controls, were positive for CK AE1/3, CK7, and p16 (strong cytoplasmic staining), and negative for CK20, GATA-3, estrogen receptor, and progesterone receptor (a profile arguing against urothelial, breast, and colorectal primaries) GATA-3: GATA-binding factor 3

Diagnostic paracentesis was performed on the day of admission, and cytologic examination of the ascitic fluid demonstrated metastatic carcinoma. Immunoperoxidase stains performed on cell block preparations, with reactive controls, showed the neoplastic cells to be positive for CK AE1/3, CK7, and p16 (strong cytoplasmic staining), and negative for CK20, GATA-binding factor 3, estrogen receptor (0%), and progesterone receptor (0%) (Figure 2). This immunoprofile argued against urothelial, breast, and colorectal primaries; PAX8 and WT1 were not performed on this specimen.

Tumor marker evaluation revealed elevated cancer antigen (CA)-125 at 445 U/mL and carcinoembryonic antigen (CEA) at 3.66 ng/mL, yielding a CA-125:CEA ratio of 121.6, strongly suggesting a gynecologic primary malignancy.

Given the absence of recent chemotherapy, radiation therapy, or other identifiable triggers, spontaneous TLS secondary to advanced ovarian carcinoma was diagnosed based on the combination of imaging, immunophenotype, and tumor marker profile. The patient received aggressive intravenous hydration, rasburicase (initiated on hospital day 1), electrolyte correction, and close multidisciplinary monitoring. Renal replacement therapy was not required. Metabolic abnormalities progressively resolved, and renal function recovered, with serum creatinine normalizing to 0.89 mg/dL by hospital day 5.

Following stabilization, the patient was evaluated by gynecologic oncology and, on hospital day 8, initiated neoadjuvant chemotherapy with carboplatin and paclitaxel. After demonstrating a favorable clinical and radiographic response, she underwent interval cytoreductive surgery approximately five months later, consisting of total abdominal hysterectomy, bilateral salpingo-oophorectomy, omentectomy, and resection of residual disease.

**Figure 3:**
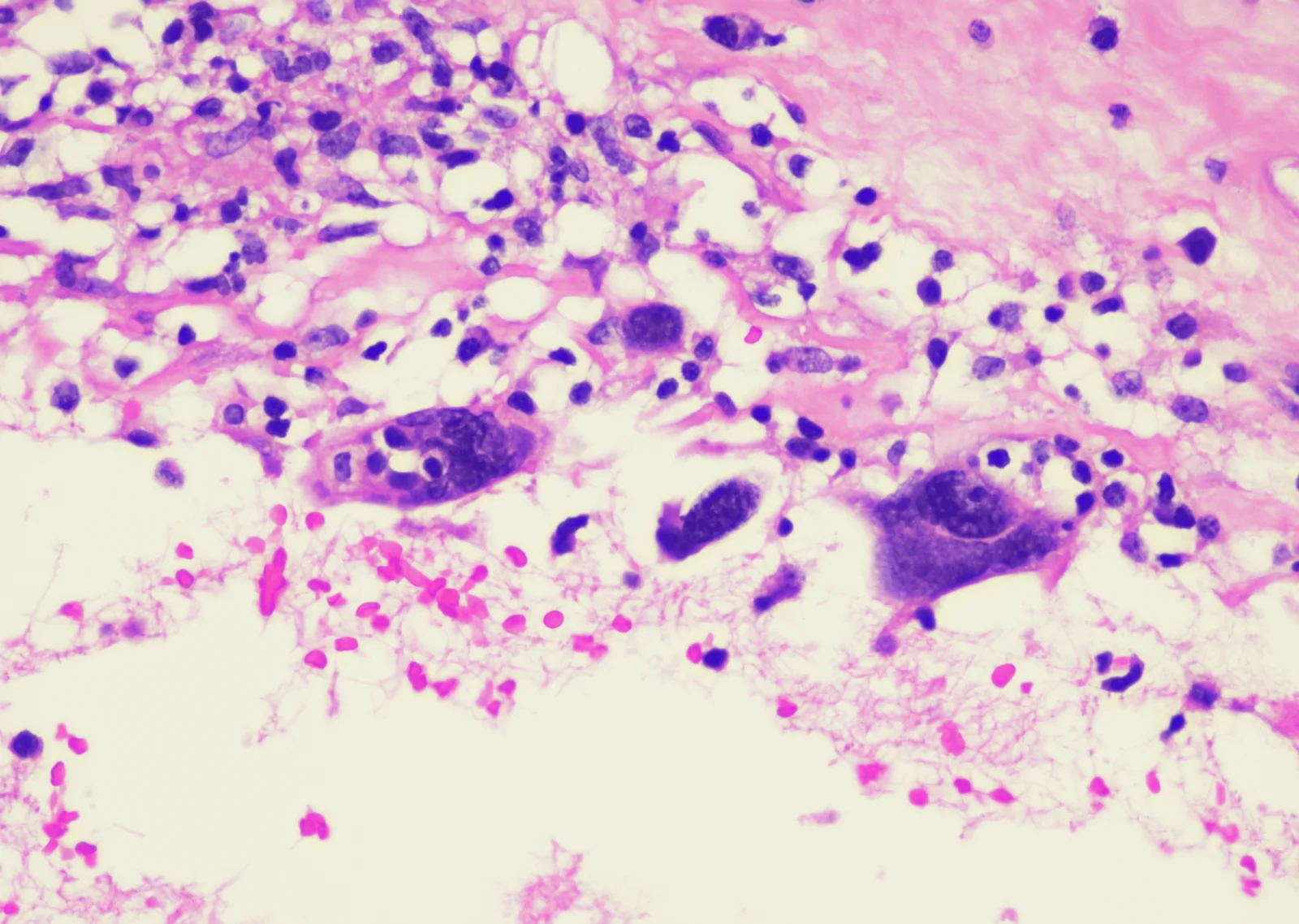
Histopathologic examination of the resected ovarian specimen (H&E stain, 40× magnification; image provided by HRP Laboratory), demonstrating extensive treatment-related changes, including chronic inflammation, stromal degeneration, and multinucleated giant cell reaction. No residual invasive carcinoma was identified, findings consistent with a complete pathologic response following neoadjuvant carboplatin-paclitaxel chemotherapy H&E: hematoxylin and eosin; HRP: Hato Rey Pathology

Histopathologic examination of the surgical specimen revealed extensive treatment-related changes characterized by ovarian necrosis, hemorrhage, hemosiderin deposition, chronic inflammation, and multinucleated giant cell reaction (Figure 3).

No residual invasive carcinoma was identified in the ovarian, omental, mesenteric, pelvic ligament, or peritoneal biopsies. These findings were consistent with an essentially complete pathologic response following neoadjuvant carboplatin-paclitaxel chemotherapy.

**Figure 4:**
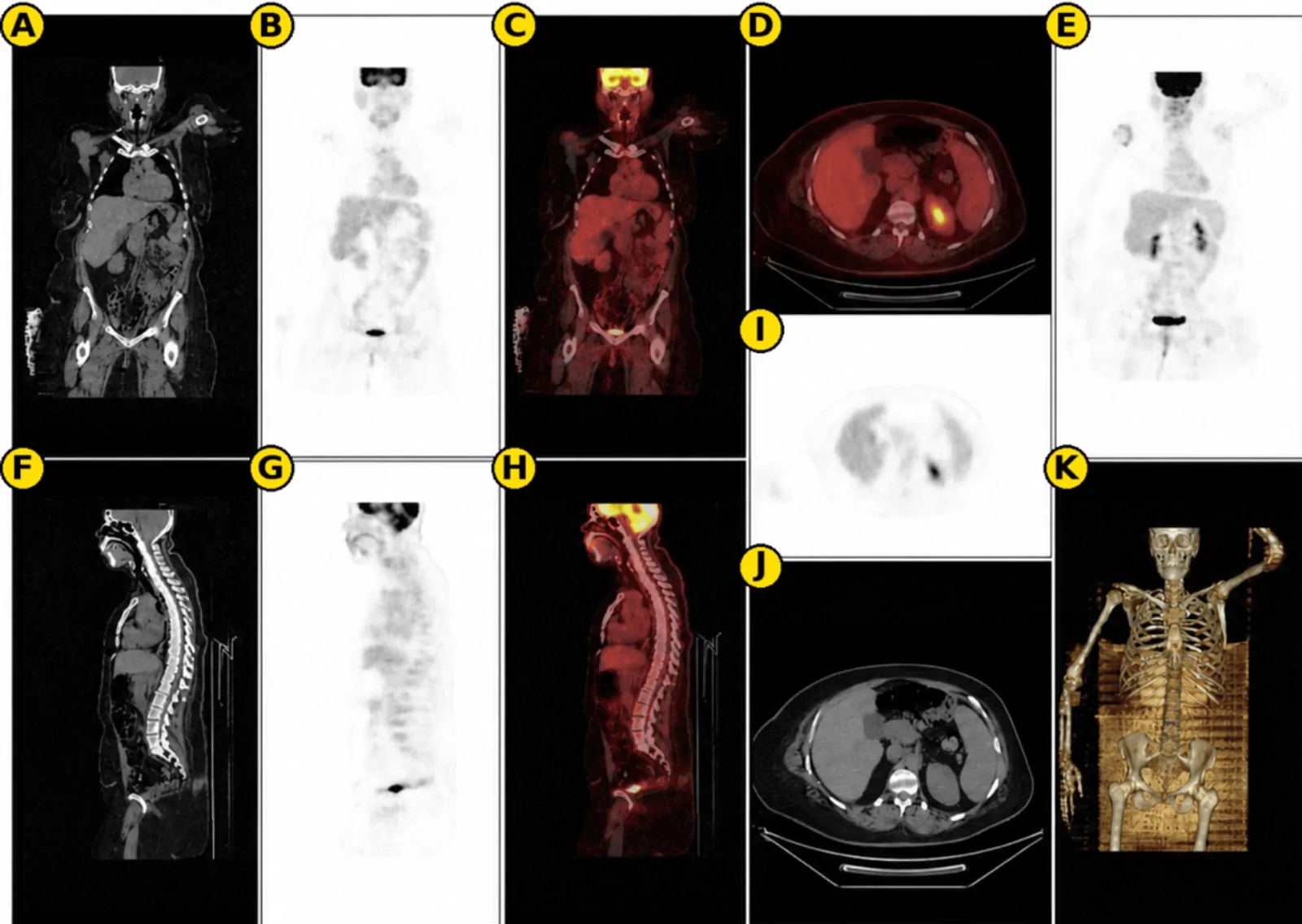
Follow-up FDG PET/CT demonstrating complete metabolic response (A) Coronal CT, bone window. (B) Coronal PET (whole-body MIP), anterior projection. (C) Coronal fused PET/CT. (D) Axial fused PET/CT at the level of the previously identified pelvic mass, showing physiologic renal radiotracer excretion with no abnormal FDG uptake. (E) Coronal PET (whole-body MIP), posterior projection. (F) Sagittal CT, bone window. (G) Sagittal PET. (H) Sagittal fused PET/CT. (I) Axial PET at the same level, showing physiologic renal radiotracer excretion. (J) Axial CT, soft-tissue window, at the same level. (K) 3D volume-rendered bone reconstruction Follow-up PET/CT performed in March 2026, demonstrating complete resolution of the previously identified pelvic mass with no evidence of FDG-avid residual or metastatic disease CT: computed tomography; PET: positron emission tomography; MIP: maximum intensity projection; FDG: fluorodeoxyglucose; 3D: three-dimensional

Subsequent surveillance demonstrated normalization of CA-125 to 5.2 U/mL. Follow-up positron emission tomography (PET)/CT imaging revealed complete resolution of the previously identified pelvic mass and no evidence of fluorodeoxyglucose-avid residual or metastatic disease (Figure 4).

At her most recent follow-up visit, the patient remained in complete clinical and radiographic remission without evidence of disease recurrence, while continuing maintenance therapy with bevacizumab biosimilars (Mvasi (bevacizumab-awwb) and Zirabev (bevacizumab-bvzr), per the institutional formulary).

## Discussion

TLS is a well-recognized oncologic emergency resulting from rapid destruction of malignant cells and the subsequent release of intracellular contents into the circulation [1,2]. The resulting metabolic abnormalities, including hyperuricemia, hyperkalemia, hyperphosphatemia, and secondary hypocalcemia, may lead to acute kidney injury, cardiac arrhythmias, seizures, multiorgan dysfunction, and death. Although TLS is frequently encountered following initiation of cytotoxic therapy in hematologic malignancies, spontaneous TLS occurring in the absence of anticancer treatment remains uncommon, particularly among patients with solid tumors [4-6].

The pathophysiology of spontaneous TLS is not completely understood. Proposed mechanisms include rapid tumor proliferation exceeding vascular supply, spontaneous tumor necrosis, extensive tumor burden, impaired renal clearance of metabolic byproducts, and increased cellular turnover [9,10]. These factors may result in the abrupt release of potassium, phosphate, and nucleic acid metabolites, producing the characteristic metabolic abnormalities observed in TLS. Unlike treatment-related TLS, spontaneous TLS may be difficult to recognize because patients frequently present before a malignancy has been diagnosed [4].

Among solid tumors, spontaneous TLS has been reported in small-cell lung cancer, hepatocellular carcinoma, breast cancer, germ cell tumors, colorectal cancer, and metastatic melanoma [4,5,9]. Reports involving gynecologic malignancies remain exceptionally rare [11]. Ovarian carcinoma is not traditionally considered a high-risk malignancy for TLS; however, advanced disease with extensive tumor burden, bulky pelvic masses, carcinomatosis, or massive ascites may create conditions favorable for spontaneous cellular breakdown and metabolic collapse [4,9].

Our patient presented with several classic risk factors associated with spontaneous TLS, including a bulky pelvic mass measuring more than 16 cm, malignant ascites, metastatic disease on cytologic evaluation, and severe metabolic derangements accompanied by acute kidney injury [4]. The absence of chemotherapy exposure or other recognized triggers strongly supported the diagnosis of spontaneous TLS. Notably, these findings developed before exposure to chemotherapy, radiation therapy, corticosteroids, or any other recognized trigger for treatment-related TLS. The absence of alternative explanations, together with fulfillment of Cairo-Bishop criteria, strongly supported the diagnosis of spontaneous TLS.

The diagnosis of ovarian carcinoma was initially established through integration of clinical, radiographic, cytologic, and serologic findings. Tumor marker evaluation demonstrated a CA-125 level of 445 U/mL and a CA125:CEA ratio of 121.6. According to contemporary gynecologic oncology practice and National Comprehensive Cancer Network recommendations, a markedly elevated CA125:CEA ratio may support a Müllerian primary when tissue acquisition is limited or when neoadjuvant treatment must be initiated before definitive surgical staging [8]. In this setting, the combination of malignant ascites, elevated CA-125, and a large pelvic mass strongly favored advanced ovarian carcinoma.

One of the most important aspects of this case was the prompt recognition of TLS despite the absence of a known malignancy. Initial symptoms, including abdominal distension, dyspnea, and oliguria, were nonspecific and could easily have been attributed solely to massive ascites or obstructive processes. However, recognition of the characteristic metabolic abnormalities led to early initiation of intravenous hydration, rasburicase therapy, electrolyte management, and close monitoring. These interventions resulted in rapid improvement of renal function and correction of metabolic abnormalities without requiring renal replacement therapy.

Acute kidney injury is among the most serious complications of TLS and is a major contributor to mortality [4,12-14]. In previously reported cases of spontaneous TLS associated with solid tumors, progression to dialysis-dependent renal failure has been frequently described [5,6]. In contrast, our patient experienced complete recovery of renal function, with serum creatinine improving from 4.09 mg/dL at presentation to 0.89 mg/dL within five days. Early recognition and administration of rasburicase likely played a critical role in preventing irreversible kidney injury [2,3].

The subsequent oncologic course further distinguishes this case from previously reported cases of spontaneous TLS in solid tumors. Following neoadjuvant carboplatin-paclitaxel chemotherapy and interval cytoreductive surgery, pathology demonstrated a complete pathologic response. Follow-up PET/CT demonstrated no evidence of metabolically active residual or metastatic disease, and CA-125 normalized to 5.2 U/mL. This favorable outcome contrasts with the historically poor prognosis associated with spontaneous TLS in solid tumors [4,9].

The favorable outcome observed in this patient contrasts with the historically poor prognosis associated with spontaneous TLS in solid tumors. Published series have reported mortality rates substantially higher than those observed in treatment-related TLS, largely because diagnosis is often delayed and patients frequently present with advanced disease and severe metabolic disturbances. This case illustrates that aggressive supportive management combined with timely multidisciplinary oncologic care can result not only in recovery from TLS but also in excellent long-term oncologic outcomes.

## Conclusions

Spontaneous TLS should be considered in patients presenting with unexplained acute kidney injury and characteristic metabolic abnormalities, even in the absence of a previously established cancer diagnosis. Although uncommon, advanced ovarian carcinoma, diagnosed here through integrated clinical, radiographic, immunohistochemical, and serologic findings, may rarely present with spontaneous TLS before initiation of anticancer therapy.

In this patient, early recognition, prompt administration of urate-lowering therapy, aggressive supportive care, and coordinated multidisciplinary management were associated with reversal of life-threatening metabolic complications and allowed progression to definitive oncologic treatment. At approximately seven months after cytoreductive surgery (from October 2025 to May 2026), the patient remained in complete clinical and radiographic remission. While causality cannot be established from a single case, this favorable course suggests that early diagnosis and treatment may support both renal and oncologic recovery in this rare presentation.
